# Simvastatin impairs murine melanoma growth

**DOI:** 10.1186/1476-511X-9-142

**Published:** 2010-12-16

**Authors:** Giovani M Favero, Michel F Otuki, Karen A Oliveira, Milton S Bohatch, Primavera Borelli, Francisco E Barros, Durvanei A Maria, Daniel Fernandes, Sergio P Bydlowski

**Affiliations:** 1 State University of Ponta Grossa, Biological and Health Science Multidisciplinary Laboratory, Ponta Grossa, Brazil; 2 Laboratory of Genetics and Molecular Hematology, Department of Hematology (LIM 31), University of São Paulo Medical School, São Paulo, Brazil; 3 Experimental Hematology Laboratory, Faculty of Pharmaceutical Sciences, Department of Clinical and Toxicological Analyses, University of São Paulo, São Paulo, Brazil; 4 Biochemistry and Biophisics Laboratories, Butantan Institute, São Paulo, Brazil

## Abstract

**Background:**

Statins induces cell cycle arrest, apoptosis, reduction of angiogenic factors, inhibition of the endothelial growth factor, impairing tissue adhesion and attenuation of the resistance mechanisms. The aim of this study was evaluate the anti-tumoral activity of simvastatin in a B16F10 melanoma-mouse model.

**Methods:**

Melanoma cells were treated with different concentrations of simvastatin and assessed by viability methods. Melanoma cells (5 × 10^4^) were implanted in two month old C57Bl6/J mice. Around 7 days after cells injection, the oral treatments were started with simvastatin (5 mg/kg/day, p.o.). Tumor size, hematological and biochemical analyses were evaluated.

**Results:**

Simvastatin at a concentration of 0.8 μM, 1.2 μM and 1.6 μM had toxic effect. Concentration of 1.6 μM induced a massive death in the first 24 h of incubation. Simvastatin at 0.8 μM induces early cell cycle arrest in G0/G1, followed by increase of hypodiploidy. Tumor size were evaluated and the difference of treated group and control, after ten days, demonstrates that simvastatin inhibited the tumor expansion in 68%.

**Conclusion:**

Simvastatin at 1.6 μM, presented cytototoxicity after 72 h of treatment, with an intense death. *In vivo*, simvastatin being potentially useful as an antiproliferative drug, with an impairment of growth after ten days.

## Introduction

Statins are a lipid-lowering class of drugs successfully used for treating hyperlipidemia, which reduces cardiovascular mortality. Simvastatin belongs to statins family, which are competitive inhibitors of 3-hydroxy-3methylglutaryl-coenzyme A (HMG-CoA) reductase, the rate-limiting enzyme that controls conversion of HMG-CoA to mevalonate, the precursor of isoprenoid compounds such as cholesterol, dolichol and ubiquinone [1-3].

The lowering cholesterol production induces a reduction of several cellular processes including prenylation of growth-regulatory proteins, cytoplasm membranes, DNA synthesis and proliferation. Several tumors have an increased mevalonate production due to an expressive needed for cholesterol associated with a rapid growth rate [4-6].

Previous studies have shown that statins have antineoplastic effects, demonstrating that statins can induce cell cycle arrest, apoptosis, reduction of angiogenic factors, and inhibition of the endothelial growth factor, impairing tissue adhesion, inhibition of cell migration and attenuation of the resistance mechanisms [7-10].

The aim of the present study was to evaluate the antitumoral activity of simvastatin *in vitro *and *in vivo*. The B16 melanoma-mouse model was used, both *in vitro *and *in vivo*. This model have a fast growth with a high lipid production and consumption, allied to an intense inflammatory response, eventually with ulcerous skin necrosis.

## Material and methods

### Animals

*In vivo *experiments were conducted in male C57BL/6J mice (18-25 g), housed at 22 ± 2°C under a 12/12 h light/dark cycle with free access to food and water until use. The animals were acclimatized to the laboratory for at least 24 h before testing and were used only once. All animal handling and experimental procedures were conducted with prior approval of the ethics committee on animal use of the University of São Paulo Medical School Hospital Ethics Committee.

### Simvastatin solution preparation

Simvastatin, in both, compressed powders and as a lactone form were a giftly provide from Prati Donaduzzi (Toledo, Brazil).

For *in vitro *assays, simvastatin in lactone form was dissolved in 1.8 mL of ethanol, added to 19 mL of 0.1 M NaOH and then incubated for 40 min at 40°C to yield the active form, followed by the addition of 0.1 M HCl to adjust the pH to 7,4 [11].

To mice experimentations, the simvastatin compressed powders were macerated and watery solutions were made.

### Cell culture

B16F10 mice melanoma cell line was generously provided by Dr. Roger Chammas (USP, São Paulo, Brazil). Cells were routinely cultured in RPMI 1640 medium supplemented with 10% fetal bovine serum (FBS) at 37°C in a humidified atmosphere containing 5% CO_2._

Cells were treated with different concentrations of simvastatin (0.8; 1.6; 3.2 and 5.6 μM) and assessed by colorimetric method of MTT, based on the reduction of MTT to Formazan by living cells. The cells were incubated in plates of 96 wells, treated with simvastatin and MTT was added at times 24, 48 and 72 hours. To dissolve the crystals of Formazan was used dimethylsulfoxide (DMSO). Quantification was obtained by the absorbance reading at a wavelength of 450 nm to 690 nm. Cell viability was also evaluated by the method of Trypan Blue exclusion (Invitrogen, Carlsbad, CA), by direct counting in a hemocytometer [12].

### Analysis of Cell Cycle by flow cytometry

Cell cycle analysis was determined by flow cytometry at the Federal State University of Paraná. Briefly, after the different treatments, 2 × 10^6 ^cells were trypsinized, washed three times with PBS, fixed in 70% ethanol, and stained with Propidium Iodide (PI) solution. All analyses were done using a FACScalibur flow cytometer (Becton Dickinson, San Jose, CA). The red fluorescence of PI was collected through a 585/42-nm band -pass filter, and the fluorescence signals were measured in a linear scale of 1024 channels. For each sample, at least 10000 events were acquired and the data were analyzed using an appropriate software (CELLQuest, Becton Dickinson, San Jose, CA) [12].

### Melanoma cells implant

All animal's procedures were made in Butantan Institute with supervision of Dr. Durvanei Augusto Maria, in accordance with University of Sao Paulo Medical School Hospital Ethics Committee. Briefly, 5 × 10^4 ^B16F10 melanoma cells were injected subcutaneously into C57Bl/6J mice. When tumors were palpable (around 8-10 days after implanted) mice were treated with oral daily dose of 5 mg/Kg of simvastatin. Tumors were measured using a caliper and volume was estimated using the formula V = (small diameter)^2 ^× (large diameter)/¾ πR [13].

### Hematological and biochemical analyses

C57BL/6J mice, weighing about 20 g and 60 days older were used. After the experiments, blood was collected from the axillary plexus. The bone marrow was collected from the femur of the same animals. The spleen cellularities were also analyzed [14].

### Liver enzymes quantification

Taking account the importance of the liver in lipids and simvastatin metabolism, we evaluated a possible hepatotoxic effect, evaluating three enzymes associated with liver injury, the glutamic-oxalacetic transaminase (GOT), the glutamic pyruvic transaminase (ALT) and gamma glutamyl transferase(gamma-GT). The experimental procedure consists in a simple addition of the 50 μL of the serum sample to 1000 μL of commercial reagent for each tested enzyme [15]. Twenty days after the melanoma cells implant and hence ten days after sinvastatin treatment has started the blood was collect.

### Determination of Blood and Counting Reticulocytes

Blood samples were collected twenty days after tumor implant from the axillary plexus of the animals in all experimental groups, which were used to perform blood and reticulocyte count, according to the techniques used in the Laboratory of Experimental Hematology of FCF/USP [14].

### Bone marrow cellularity

The animals were first anesthetized, exsanguinated and sacrificed by cervical dislocation. The bone marrow was obtained by washing the cavity with the culture femoral McCoy's 5A modified (Sigma, Chemical Company, USA) ice cream [14].

### Myelogram

The bone marrow cells suspension samples were prepared on slides of cell spin cytocentrifuge (Incibrás) and stained by the method of May-Grünwald-Giemsa modified Rosenfeld. The cells were classified as blastic and mitotic cells, neutrophil young granulocytes (promyelocytic and myelocytes) in ring stage (metamyelocytes and rod), and segmented eosinophils, monocytes and macrophages, lymphocytes and plasma cells, pro-erythroblasts and basophilic erythroblasts, polychromatic and orthochromatic [14,16].

### Splenogram

Sleen cells were collected by the same sacrificed animals of hematological procedures. The spleen was transferred to plastic Petri dish with iced culture McCoy's 5A modified (Sigma, Chemical Company, USA) containing 10% EDTA, used to collect the cells. In the Petri dish, the spleen capsule was broken in one of its ends, with the aid of two needles bent into "L", and fixed in syringes. The cells were gently removed from the dish by the dissociation method. The suspension was homogenized in the Petri dish with standard Pasteur pipette to complete cell separation and transferred to conical plastic tube and kept in an ice bath. The cells obtained from the spleen were quantified in a Neubauer chamber hemocytometer after dilution of 1:500 liquid Turk, diluent composed of 1% solution of glacial acetic acid. For morphological evaluation, the slides were performed in cell suspension cellspin cytocentrifuge (Incibras) and subsequently submitted to the staining of May-Grunwald GIENS, modified by Rosenfeld, and peroxidase method modified by Graham Knoll, for identification and quantification of different types cell [16].

### Statistical Analysis

Tumor growth data were analyzed by Student's *t *test, survival rate data were analyzed by Kaplan-Meyer curves and hematological data were analyzed by one-way ANOVA with Tukey posttest using Graph Pad Prism Stat version 4.0 for Windows.

## Results

### In vitro effects of simvastatin on B16F10 cell growth and death

**Figure 1:**
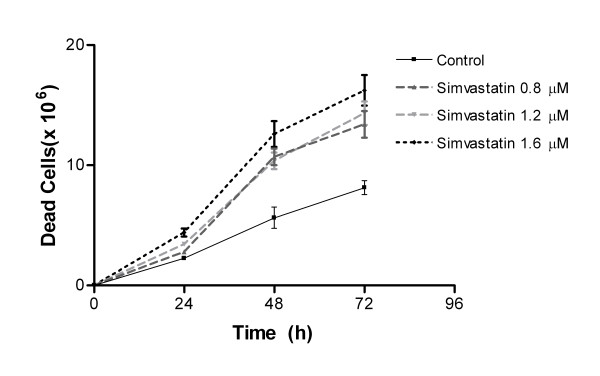
**Sinvastatin reduces the number of viable cells in cultured melanoma after 48 hours**. Cells were cultured in the presence of the statin in lactone form, or with no treatment for 4 days. Three wells of each condition were subjected to the trypan blue assay. The mean values were calculated and plotted as the percentage of the vehicle-treated. The experiment was repeated three times. Sinvastatin values are indicated by p when significantly different from controls (P < 0.05). Data are expressed as mean and standard error of the mean. Statistical analysis was performed using ANOVA followed by Tukey post hoc t test. * p < 0.05 compared with the control group.

**Figure 2:**
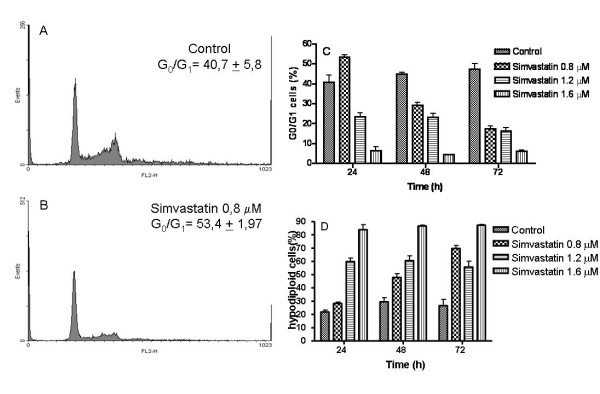
**Flow cytometry Cell cycle analysis, using propidium iodide as a DNA intercalating agent**. A) Histogram representative of Control group after 24 h of seed; B) G0/G1 cell cycle arrest induced by 0.8 μM of simvastatin. C) Percentage of G0/G1 cells in witch treatment. D) Increased cell death due to simvastatin effects evaluated by hypodiploid cells amount. Each bar represents the mean, and vertical lines are the standard error of the mean of a representative assay performed in triplicates.

The Melanoma cell line B16F10 was exposed to different amounts of simvastatin, the results evidenced that at a concentration of 0.8 μM, 1.2 μM and 1.6 μM simvastatin had toxic effect. When incubated with concentration of 1.6 μM, the toxicity was present in the first 24 h of incubation, inducing a massive death (Figure 1). Cell cycle analyses (Figure 2) shows that 0.8 μM of simvastatin induces early cell cycle arrest in G0/G1, 24 hours, followed by increase of hypodiploid cells, a characteristic of apoptosis cell death.

### Simvastatin inhibits melanoma growth in C57Bl/6J mice

To test the hypothesis that inhibiting endogenous cholesterol synthesis could limit melanoma growth *in vivo*, we administered daily oral doses of simvastatin on B16F10 melanoma bearing mice. Concentration of 5 mg/Kg/day of simvastatin were orally given to mice for 10 days, 10 days after the inoculation of the tumor cells.

**Figure 3:**
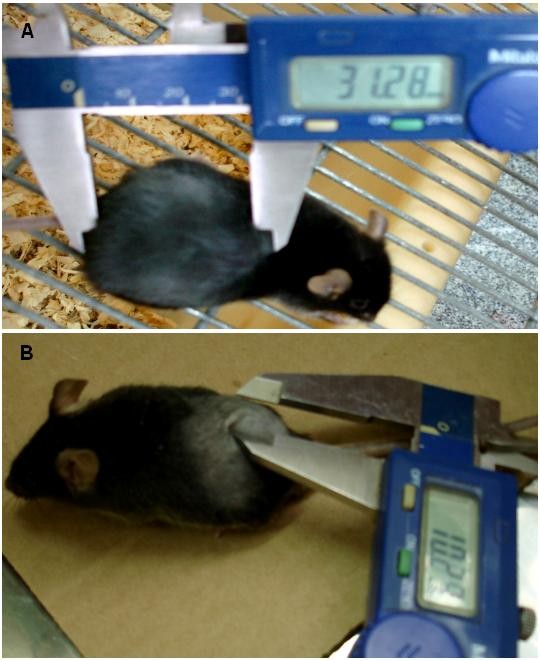
**Example of twenty days of mice bearing melanoma; A) Control group and B) Treated with high daily doses of simvastatin with notable reduction in tumor size**.

**Figure 4:**
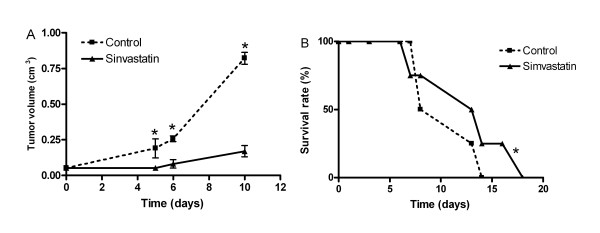
**Effect of high daily doses of simvastatin on tumor growth and survival rate of B16F10 tumor bearing mice**. C57Bl6J mice were implanted with 5·104 B16 melanoma cells subcutaneously injected in dorsal region of B57CL/6 mice (8 mice per group). After the tumors had reached approximately 4 mm in diameter (10 days) the daily treatment were started for 10 days. A) Perpendicular tumor diameters were measured daily to estimate tumor volume. Controls were saline solution in the same schedule. P values were calculated by using the Student's t test and are indicated by p when significantly different from controls (P < 0.05). B) Percent B16 melanoma bearing mice survival in response to the treatment as a function of time. The survival rates were calculated daily and the experiment was terminated when all the mice of control group died (at day 28). Survival rate data were analyzed by Kaplan-Meyer curves.

The effects of simvastatin on tumor volume are demonstrated in Figure 3 and 4. The Figure 3 exemplifies the action over tumor size after high daily doses of simvastatin compared to control mouse with same tumoral implant. As can be see in Figure 4, the 10 days follow-up effects of simvastatin treatment significantly impair tumor growth (p < 0,05). The melanoma presented a 25% of final tumor sizing compared to the controls. The Figure 4B shows the Kaplan-Meyer survival curves of mice treated with simvastatin. There are no survival mice of the control group on day 24. Twenty-five percent of the simvastatin treated mice had a survival of 28 days.

Total tumoral regression was not developed in anyone of the melanoma bearing mice, and when the pharmacologic treatment was aborted the tumor re-started to grow (data not show).

### Simvastatin did not induce liver injury

The transaminases are typically intracellular enzymes. Increased plasma concentration of both leads to the predominant diagnosis of hepatocellular injury, which may be acute as in viral hepatitis, drug-induced or autoimmune, or chronic liver disease or ischemic, and others. The gamma-GT, gamma glutamyl transferase, is present in the liver (in hepatocytes and in biliary epithelium) and in other organs such as pancreas and kidneys. Quantification of Gamma-GT is useful in evaluation of acute and chronic liver diseases, with high enzyme activity in the tables of cholestasis intra or extra-hepatic. Despite being a very sensitive marker of hepatobiliary disease, is not specific, being increased in other diseases such as diabetes mellitus and renal failure. In addition, some drugs act as inducers, especially the barbiturates, the diphenyl-hydantoin and the tricyclic antidepressants, in addition to ethanol.

Our results showed at Table 1 had a large variation being not statistically significant for GOT or Gamma-GT. The ALT showed significance between the groups treated with simvastatin and control groups. These isolated findings did not represent liver injury when analyzed globally with the other transaminase, Gamma-GT and liver histopathology (data not show).

**Table 1:** Effect of simvastatin on liver enzymes.

Groups	GOT (U/L)	ALT (U/L)	Gamma-GT (U/L)
Control	31 ± 16	19 ± 1	26 ± 11

Control-Simvastatin	28 ± 13	31 ± 9 *	38 ± 12

Tumor Control	20 ± 5	16 ± 5	21 ± 7

Tumor-Simvastatin	20 ± 4	26 ± 4 *	21 ± 7

Data are expressed as mean ± S.E.M. of seven animals per group.

* denotes significant difference from respective control at p < 0,05

### Dual effect of simvastatin over bone marrow and spleen cellularity

**Figure 5:**
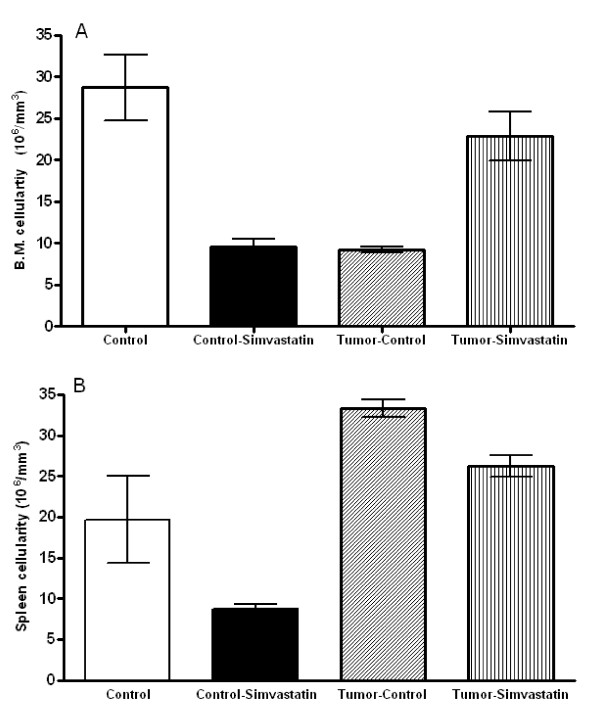
**Femoral Bone Marrow (A) and Spleen cellulartiy (B) cellularity are decreased in mice treated with simvastatin (Control-Simvastatin) but it is equal when mice bearing melanoma treated with same doses of simvastatin**. Each bar represents the mean of the data from eighth animals and the vertical lines are the standard error of the mean. Statistical analysis was performed using ANOVA followed by Tukey post hoc t test. * p < 0.05 compared with the control group.

To estimate how toxic simvastatin could be, we evaluated the cellularity of bone marrow and spleen, remembering that these are hematological producing centers in rodents. The results summarized in Figure 5 and Table 2 show that simvastatin protect or keep the myeloid tissue in a normal account on melanoma bearing mice. Interestingly, the animals without tumor that received simvastatin had a decrease on the amount of cells into the analyzed tissues.

**Table 2:** Blood cells evaluation.

Groups	Hemoglobin (g/dL)	Total Leukocytes (103/mm3)	Eosinophils (%)
Control	12.3 ± 0.75	5.2 ± 0.7	6.7 ± 1.6

Control-Simvastatin	12.8 ± 1.9	3.3 ± 0.1 *	4.7 ± 1.4

Tumor Control	8.6 ± 2.8	3.1 ± 0.2 *	2.6 ± 0.7 *

Tumor-Simvastatin	12.4 ± 1.2	3.9 ± 0.8 *	8.5 ± 3.0 *

Data are expressed as mean ± S.E.M. of seven animals per group.

* denotes significant difference from respective control at p < 0.05.

The evaluation of white blood cell count showed no significant differences in the amount of total leukocytes. The differential leukocytes count showed significant difference only for the eosinophils percentage in melanoma bearing mice treated with simvastatin. Studies such as Cormier [17] have previously shown that the increase in the number of eosinophils in solid tumors such as melanoma, is an important factor for the demonstration of persistent inflammation and on the initial tumor [17]. There are no difference in percentage of promyelocytic, plasma, total blasts, erythroblasts and megakaryocytes in all groups (data not shown).

## Discussion

No clear evidence exists that some widely prescribed cholesterol lowering drugs can decrease the risk of melanoma, a deadly and malignant skin cancer. The present study demonstrates that simvastatin, a HMG-CoA reductase inhibitor promoted a strong tumor growth-inhibiting effect over a mice melanoma model.

Although there are many reports on the anticancer properties of statins, as far we know this is the first evidence of anti-tumoral effect of simvastatin on mice melanoma *in vivo*. Recently Saito and colleagues demonstrates the effects of simvastatin over melanoma cells *in vitro*, showing the promotion of cell cycle arrest and apoptosis [18]. Our results shows a clear inhibitory effect of simvastatin on B16F10 tumor growth (approximately 75% tumor inhibition over 10 days period). These effects could be connected to diminish of endogenous cholesterol synthesis and disruption of cellular membrane stability as demonstrated by previous works [19-23].

Several reports have shown that simvasatina has anti-inflammatory effects, for instance by reducing the production of cytokines. Together these data suggest that the effect of simvastatin acts on the tumor inflammatory microenvironment [24-26].

Recently Otuki *et *al evidenced the use of topical simvastatin for a treatment of skin inflammatory condition [27]. The massive growth of melanoma cells in this model is directly dependent of an inflammatory microenvironment, cells such as tumor-infiltrating monocytes/macrophages, neutrophils, mast cells, eosinophils and activated T lymphocytes contribute to malignancies by releasing growth and survival factors, as well as extracellular proteases, proangiogenic factors and chemokines [28,29].

An extensive review of the potential of statins on cancer treatment elucidate the know mechanisms involved in tumor impairment due to these drugs: a) cell cycle arrest allied to up regulation of cell-cycle inhibitors b) apoptosis, intrinsic, mediated principally by mitochondria pathway, or extrinsic throw death ligands increase; c) reduction of pro-angiogenic factors, inhibition of endothelial cell growth, impairment of endothelial adhesion; d) reduction of adhesion molecules, inhibition of tumor cell migrating factors; e) attenuation of resistance mechanisms. Hematological analysis indicated that simvastatin treatment performed maintenance reference value of circulating blood cells. Bone marrow and spleen analysis also showed a protect effect of simvastatin in melanoma-bearing mice. Surprising, the mice without melanoma implant that received simvastatin had a diminish velour both in circulating blood cells, in bone marrow and spleen. Our suggested hypothesis is coupled to the high tumor vascularization that led to a concentrated amount of compounds such as simvastatin [29].

## Competing interests

The authors declare that they have no competing interests.

## Authors' contributions

GMF conceived the study, participated in its design and coordination. MFO carried out the cytometric assays. KAO and MSB worked with the cellular experiments. PB and FEB participated in hematology and biochemistry procedures. DAM has worked extensively with the testing of dorsal melanoma mice. DF participated intensely in the discussion of results and drafting the manuscript. SPB worked as team leader, organizer and tutor of this work. All authors read and approved the final manuscript.
